# Associations between physical activity, left atrial size and incident atrial fibrillation: the Tromsø Study 1994–2016

**DOI:** 10.1136/openhrt-2021-001823

**Published:** 2022-01-24

**Authors:** Kim Arne Heitmann, Maja-Lisa Løchen, Michael Stylidis, Laila A Hopstock, Henrik Schirmer, Bente Morseth

**Affiliations:** 1 School of Sport Sciences, UiT The Arctic University of Norway, Tromsø, Norway; 2 Centre for Research and Education, University Hospital of North Norway, Tromsø, Norway; 3 Department of Community Medicine, UiT The Arctic University of Norway, Tromsø, Norway; 4 Department of Cardiology, Akershus University Hospital, Lørenskog, Norway; 5 Institute of Clinical Medicine, University of Oslo, Oslo, Norway; 6 Department of Clinical Medicine, UiT The Arctic University of Norway, Tromsø, Norway

**Keywords:** arrhythmias, cardiac, echocardiography, epidemiology

## Abstract

**Aims:**

Left atrial (LA) enlargement is an independent risk factor for atrial fibrillation (AF). Interestingly, some athletes have increased risk of AF, which may be linked to LA enlargement; however, little is known about the relationship between LA enlargement and AF risk at moderate-level physical activity (PA). We aimed to explore the associations between PA, LA size and risk of incident AF, and if PA can attenuate the risk of AF with LA enlargement.

**Methods:**

This prospective study followed 2479 participants (52.4% female), free from known cardiac pathology, for median 20.2 years. Participants were followed up for hospital-diagnosed AF, confirmed by electrocardiography, from 1994-95 through 2016. At baseline, LA size was evaluated by anteroposterior LA diameter, and PA was self-reported by questionnaire.

**Results:**

We observed a U-shaped relationship between PA and AF, and moderately active had 32% lower AF risk than inactive (HR_adjusted_ 0.68, 95% CI 0.50 to 0.93). Participants with LA enlargement had 38% higher AF risk compared with participants with normal LA size (HR_adjusted_ 1.38, 95% CI 1.12 to 1.69). However, the increased AF risk with LA enlargement was attenuated by PA; compared with inactive participants with LA enlargement, the AF risk was 45% lower among active with LA enlargement (HR_adjusted_ 0.55, 95% CI 0.39 to 0.79). AF risk in active participants with LA enlargement did not differ from active with normal LA size. These patterns were observed in both men and women, and in participants over/under 65 years.

**Conclusion:**

Moderate PA was associated with reduced AF risk, and PA attenuated the increased risk of AF with LA enlargement in both men and women and all age groups.

**Key questions**

**What is already known about this subject?**

- Left atrial (LA) enlargement is an independent risk factor for atrial fibrillation (AF).
- Moderate physical activity (PA) is associated with reduced risk of cardiovascular disease including AF.
- Little is known about the role of moderate PA in the association between LA size and risk of AF in the general population.

**What does this study add?**

- In this prospective study, PA was associated with lower risk of AF in a U-shaped pattern.
- Participants with LA enlargement had higher AF risk compared with participants with normal LA size.
- PA attenuated the increased risk of AF with LA enlargement in both men and women and all age groups.

**How might this impact on clinical practice?**

- We suggest that the protective effect of moderate PA outweighs the potential risk of AF with LA enlargement.
- Increased understanding of the relationship between PA and AF may guide clinicians in implementing PA in primary and secondary prevention of AF.

## Introduction

Atrial fibrillation (AF) is the most common clinical arrhythmia worldwide, associated with considerably morbidity, such as stroke and heart failure.^1^ In 2016, the global prevalence of AF was 43.6 million, and lifetime risk for developing AF in Europeans over 55 years is approximately one in three.^1^ Furthermore, the risk of AF increases with ageing, and due to increased longevity in the general population, a twofold increase in AF is expected during the next decades.^1^ Thus, more knowledge on modifiable risk factors for prevention of AF is needed.

Physical activity (PA) is recommended for health promotion and disease prevention,^2^ and it is well documented that PA is associated with reduced risk of cardiovascular disease including AF^1 3^ and all-cause mortality.^4^ Paradoxically, studies have reported that high amounts of vigorous exercise may attenuate the benefits of moderate PA, or even increase the risk of AF.^5 6^ Studies have demonstrated that the risk of AF increases with high levels of lifelong exercise, or participation in endurance sports,^7 8^ and that elite athletes more frequently experience AF than non-athletes.^9^

Several studies have reported that left atrial (LA) enlargement is an independent risk factor for AF in the general population,^8 10 11^ and pathological conditions that increase LA size (eg, hypertension, valvular heart disease and heart failure) further increase the risk of AF.^12^ In addition to increased risk of AF, LA enlargement is a risk factor for stroke and all-cause mortality^13 14^ in the general population. Additionally, LA size increases with exercise,^15^ and studies have observed that LA size is larger in both athletes^16^ and in active adult and elderly individuals.^17^ Some elite athletes even have LA enlargement overlapping LA size seen in cardiac pathology.^18^ The mechanisms underlying the association between exercise and AF are not established, but it is suggested that the increased risk of AF with extensive endurance exercise may be linked to LA enlargement.^19^

However, despite increased risk of AF with both LA enlargement and with vigorous exercise, convincing data linking the combination of exercise and LA size to AF are lacking and are largely speculative.^19 20^ Furthermore, studies have demonstrated that LA function is preserved,^18^ or even improved in athletes with LA enlargement.^15^ Also, LA enlargement has been reported in endurance athletes, without correlation with electrophysiologic remodelling.^21^ Thus, it is of importance to elucidate if LA enlargement is a benign physiological adaption of PA and exercise, or part of a pathophysiological mechanism increasing the risk of AF.

Current knowledge of the relationship between exercise, LA remodelling and AF is mainly derived from studies of athletes. Thus, little is known about the role of moderate PA in the association between LA size and risk of AF in the general population. Therefore, our objective was to explore the associations between PA, LA size and incident risk of AF in a general adult and elderly population. Furthermore, we aimed to explore if PA attenuates the increased risk of AF seen with LA enlargement.

## Methods

### Study population

The Tromsø Study is a single-centre population-based cohort study with seven repeated health surveys of the population of the Tromsø municipality, Norway.^22^ The present study includes participants from the fourth survey of the Tromsø Study (Tromsø4, 1994–1995), with 22 years follow-up for AF until 31 December 2016.

In Tromsø4, all inhabitants ≥25 years were invited (n=37 558), with 27 158 men and women attending the first visit (77% attendance). Of these, all men aged 55–74 years, all women aged 50–74 years and 5%–8% random samples of the other age groups aged <85 years were invited to a second visit with extended examinations. Of these, a total of 7965 participants (76% of the 10 542 invited) attended the second visit. These participants were alternately allocated by computer to one of two lines of examination when attending the first visit, and 3287 participants in one of the lines were examined by echocardiography. These participants did not differ from the total sample attending the second visit.^23^

In total, 2714 participants provided valid data on PA in combination with valid echocardiography data and status of myocardial infarction at baseline. We excluded participants with previous or present documented AF (n=45), valvular heart disease (n=27), left ventricular (LV) ejection fraction <40% (n=13), previous myocardial infarction (n=129) and LA below normal reference range (n=16) at baseline. Furthermore, one participant was excluded due to missing data on a covariate (systolic blood pressure). Finally, our analytical sample consisted of 2479 participants free from known cardiac pathology, with valid data on PA, AF, echocardiography and covariates at baseline (table 1).

**Table 1:** Baseline and follow-up characteristics stratified by PA: the Tromsø Study 1994–1995

	Inactive (n=1502)	Active (n=977)	Total (n=2479)
**Baseline characteristics**			
Age, years	60.8 (9.3)	55.1 (11.9)	58.6 (10.7)
Sex, % (n) female	61.6 (925)	38.2 (373)	52.4 (1298)
Body mass index, kg/m^2^	25.9 (4.0)	25.5 (3.3)	25.8 (3.8)
Systolic blood pressure, mm Hg	145.1 (22.3)	140.0 (20.5)	143.1 (21.8)
Diastolic blood pressure, mm Hg	83.2 (12.5)	81.7 (12.3)	82.6 (12.4)
Heart rate rest, beats/min	71.7 (12.1)	69.0 (12.1)	70.6 (12.2)
LDL cholesterol, mmol/L	5.2 (1.3)	4.9 (1.3)	5.1 (1.3)
C-reactive protein, mg/L	2.7 (6.1)	2.3 (7.1)	2.6 (6.5)
Antihypertensives, % (n)	12.3 (184)	7.1 (69)	10.3 (253)
Hypertension, controlled, % (n)	1.9 (29)	1.3 (13)	1.7 (42)
Hypertension, uncontrolled, % (n)	10.3 (155)	5.7 (56)	8.5 (211)
Hypertension, untreated, % (n)	46.5 (699)	42.0 (410)	44.7 (1109)
Stroke, % (n)	2.1 (31)	1.1 (11)	1.7 (42)
Diabetes, % (n)	2.7 (40)	1.4 (14)	2.2 (54)
Palpitations, % (n)	24.4 (309)	20.5 (185)	22.8 (494)
Thyroid disease, % (n)	5.4 (81)	3.9 (38)	5.5 (119)
Smoking, % (n)	33.6 (504)	27.7 (271)	31.3 (775)
Alcohol, glasses/14 days	3.6 (5.9)	5.2 (7.0)	4.2 (6.5)
Coffee, cups/day	5.4 (3.2)	5.5 (3.6)	5.5 (3.4)
LV mass index, g/h^2.7^	44.4 (12.3)	42.9 (12.1)	43.8 (12.2)
LV mass index, g/h^2.7^ female	43.6 (12.0)	39.4 (10.5)	42.4 (11.8)
LV mass index, g/h^2.7^ male	45.7 (12.6)	45.0 (12.5)	45.4 (12.5)
LA diameter, cm	3.9 (0.6)	4.0 (0.6)	4.0 (0.6)
LA diameter, cm female	3.8 (0.6)	3.7 (0.5)	3.8 (0.5)
LA diameter, cm male	4.1 (0.6)	4.2 (0.5)	4.2 (0.6)
LA diameter index, cm/m^2^	2.2 (0.3)	2.1 (0.3)	2.2 (0.3)
LA diameter index, cm/m^2^ female	2.2 (0.3)	2.2 (0.3)	2.2 (0.3)
LA diameter index, cm/m^2^ male	2.1 (0.3)	2.1 (0.3)	2.1 (0.3)
Physical activity *			
Inactive, % (n)	100 (1502)	0.0 (0)	60.6 (1502)
Low, % (n)	0.0 (0)	39.2 (383)	15.4 (383)
Moderate, % (n)	0.0 (0)	40.0 (391)	15.8 (391)
Vigorous, % (n)	0.0 (0)	20.8 (203)	8.2 (203)
**Follow-up characteristics**			
AF during follow-up, % (n)	17.9 (269)	13.3 (130)	16.1 (399)
Follow-up time, years	15.9 (6.7)	17.6 (6.2)	16.6 (6.6)

Numbers are mean±SD or percentage and n.

*Hard leisure time PA, weekly average over the last year.

AF, atrial fibrillation; LA, left atrial; LDL, low-density lipoprotein; LV, left ventricular; PA, physical activity.

### Assessment of PA

PA was assessed by a validated questionnaire measuring duration of light and hard leisure time PA,^24^ which has been used in the Norwegian health surveys constituting The Cohort of Norway.^25^ The PA questionnaire was used to assess the weekly average hours with light (not sweating or out of breath) and hard leisure time PA (sweating/out of breath), respectively, over the last year. In this study, we used the question about hard PA in the main analyses: (1) 0 hours/week (inactive), (2) 0–1 hour/week (low), (3) 1–2 hours/week (moderate), (4) ≥3 hours/week (vigorous). Time spent going to/from work was considered as leisure-time PA. In some analyses, hard leisure-time PA is dichotomised into inactive (0 hours/week; option 1) and active (>0 hours/week; option 2–4).

For sensitivity analysis, we combined questions about light and hard PA into five categories: (1) light and hard PA 0 hours/week (sedentary), (2) light PA >0 hours/week and hard PA 0 hours/week (inactive), (3) hard PA 0–1 hour/week (low), (4) hard PA 1–2 hours/week (moderate), (5) hard PA ≥3 hours/week (vigorous).

### Cardiac structure and function

Echocardiography was performed by three medical doctors (one trained in echocardiography and two expert cardiologists), using a VingMed CFM 750 ultrasound scanner (VingMed Sound A/S, Horten, Norway). The echocardiographic assessment was performed with the use of standard imaging planes in a supine left lateral position. The echocardiographic assessment was performed online in one heart cycle, but remeasured if deviating from eye-balled estimates. Procedures and details of echocardiographic assessments in Tromsø4 are described elsewhere.^23^

LA anteroposterior diameter was measured at the end of the LV systole by M-mode echocardiography in the parasternal short axis view at the aortic valve level, after alignment of LV in long axis view, using the leading edge-to-leading edge convention.^26^ LA diameter was indexed to body surface area^27^ and presented as centimetres per metre squared (cm/m^2^). LA diameter index was categorised into small (<1.5 cm/m^2^), normal (1.5–2.3 cm/m^2^) and enlarged (≥2.3 cm/m^2^).^26^ LV dimensions were measured at the end of diastole and systole, in the parasternal short axis view, after alignment of LV in long axis view to the leading edge-to-edge convention.^26^ LV ejection fraction was calculated by the Teichholz formula,^28^ using end-diastolic and end-systolic LV dimensions. LV myocardial mass was calculated according to the cube formula^26^ and further indexed to height raised to the allometric power of 2.7.^29^

Valvular heart disease was defined by of the following criteria: (a) mitral stenosis identified by pulsed Doppler, (b) aortic stenosis (peak gradient >36 mm Hg) by continuous Doppler, (c) mitral regurgitation (regurgitant jet area >7 cm^2^) by two-dimensional colour Doppler imaging and/or (d) aortic regurgitation (vena contracta width >50% of LV outflow tract, or colour jet exceeding mitral valve coaptation point), by colour M-mode.

A reproducibility study was performed by the two main observers in a subsample of 49 subjects.^23^ Intraobserver differences (mean±SD) for LV mass were 3.0±39.0 g and 7.0±25.5 g, respectively, whereas interobserver difference was 14.8±32.5 g.

### Covariates

Baseline data from Tromsø4 include the following covariate extracted from self-reported questionnaires, physical examinations and blood samples: daily cigarette smoking (yes/no), coffee consumption (cups/day), diabetes (yes/no), use of antihypertensives (currently or previously/never), myocardial infarction (previously/no), stroke (previously/no), thyroid disease (yes/no) and palpitations (yes/no). Alcohol consumption was summarised from three questions reporting number of glasses of beer, wine or spirit normally consumed within a 14-day period.

Heart rate and blood pressure were recorded three times with 1 min intervals after 2 min seated rest, by specially trained nurses using an automatic device (Dinamap Vital Signs 1846 monitor, Criticon, Florida). For resting heart rate, the lowest recorded reading was used. For blood pressure, the average from reading two and three was used. Blood pressure was classified into hypertension groups: (a) normotensive (systolic blood pressure <140 mm Hg, diastolic blood pressure <90 mm Hg and no self-reported use of antihypertensives), (b) hypertensive, controlled (systolic blood pressure <140 mm Hg, diastolic blood pressure <90 mm Hg and self-reported use of antihypertensives), (c) hypertensive, uncontrolled (systolic blood pressure ≥140 mm Hg and/or diastolic blood pressure ≥90 mm Hg and self-reported use of antihypertensives) or (d) hypertension, untreated (systolic blood pressure ≥140 mm Hg and/or diastolic blood pressure ≥90 mm Hg and no self-reported use of antihypertensives). Height and weight were measured with participants wearing light clothing and no shoes. Body mass index was calculated as weight (kg) divided by height squared (m^2^).

Blood samples were analysed for total and high-density lipoprotein cholesterol (mmol/L) and C-reactive protein (mg/L) at the Department of Clinical Chemistry, University Hospital of North Norway. Low-density lipoprotein (LDL) cholesterol was estimated by subtracting high-density lipoprotein cholesterol from total cholesterol.

### Follow-up and detection of AF

The participants were followed from the date of examination in 1994–95 (Tromsø4), until (a) the date of first documented AF, (b) date of censoring due to death or migration or (c) end of follow-up on 31 December 2016, whichever came first.

The follow-up data on AF were derived from the diagnosis registry of the University Hospital of North Norway, the only hospital in the region, by linking the hospital records of AF to the participants’ unique Norwegian national 11-digit identification number. Hospital records were searched for incident cases of AF confirmed by electrocardiography confirmation. Additionally, in participants with diagnoses of cerebrovascular or cardiovascular events, but without diagnosis of arrhythmias, hospital records were manually searched for notes on AF. Details of ascertainment of AF are described elsewhere.^30^ The events were evaluated and adjudicated by an independent expert endpoint committee. Postoperative AF within 28 days after surgery, myocardial infarction or other acute cardiac events, as well as AF documented the last week of life, were all classified as non-cases.

### Statistical methods

Characteristics of the study population are presented as means with SD or percentages with number of observations (n). Associations between PA, LA size and AF risk were evaluated using univariable and multivariable Cox proportional hazard regression analyses. The associations are presented as HRs with 95% CI. The proportional hazard assumption was confirmed by visual inspection of log-minus-log plots. Model 1 was unadjusted, model 2 was adjusted for age, sex, body mass index and systolic blood pressure.

To test the robustness of the fully adjusted model 2, we performed sensitivity analyses with additional covariates (smoking, coffee consumption, diabetes, LDL cholesterol, palpitations, LV myocardial mass index, thyroid disease, C reactive protein, alcohol consumption and resting heart rate) stepwise, or jointly, added to the model.

In the fully adjusted model 2, we tested for possible interactions between PA*sex, PA*age, PA*hypertension groups, PA*body mass index and PA*LA size in a stepwise analysis of the association between PA and AF. We observed a significant interaction between PA*LA size (p=0.023), whereas no significant interactions were indicated for the other interaction terms (p>0.50). However, given previously reported age and sex differences in the association between PA and AF risk, we also stratified our main analyses by sex and age in addition to LA size.

All statistical analyses were performed using SPSS V.26 (SPSS, Illinois, USA), with a two-sided p≤0.05 considered statistically significant.

## Results

In total, 1181 men (57.7±10.7 years) and 1298 women (59.3±10.7 years), 25–83 years, were included in our analyses. Of these, 399 participants (214 men and 185 women) were diagnosed with AF during a mean follow-up of 16.6±6.6 years (median 20.2 years, IQR 11.6–21.9 years). Descriptive characteristics for the participants stratified by level of PA are presented in table 1.

Overall, we observed 32% lower risk of AF in moderately active than in inactive participants (HR_adjusted_ 0.68, 95% CI 0.50 to 0.93), and a U-shaped relationship was observed (table 2). Furthermore, participants with LA enlargement had 38% higher risk of AF compared with participants with normal LA size (HR_adjusted_ 1.38, 95% CI 1.12 to 1.69).

**Table 2:** Association between PA and AF (HR ±95% CI): the Tromsø Study 1994–1995

	N (n=2479)	AF events, % (n)	Person-years (mean±SD)	Model 1, HR (95% CI)	Model 1, P-value	Model 2, HR (95% CI)	Model 2, P-value
Inactive	1502	17.9 (269)	15.9 (6.7)	1.00 (ref.)		1.00 (ref.)	
Low	383	13.1 (50)	17.4 (6.5)	0.64 (0.47 to 0.87)	0.004	0.80 (0.59 to 1.09)	0.150
Moderate	391	12.3 (48)	17.8 (6.0)	0.59 (0.43 to 0.80)	0.001	0.68 (0.50 to 0.93)	0.017
Vigorous	203	15.8 (32)	17.7 (6.1)	0.76 (0.53 to 1.09)	0.138	0.87 (0.60 to 1.27)	0.473

Model 1 was unadjusted. Model 2 was adjusted for age, sex, body mass index and systolic blood pressure.

AF, atrial fibrillation; PA, physical activity; ref., reference.

**Figure 1:**
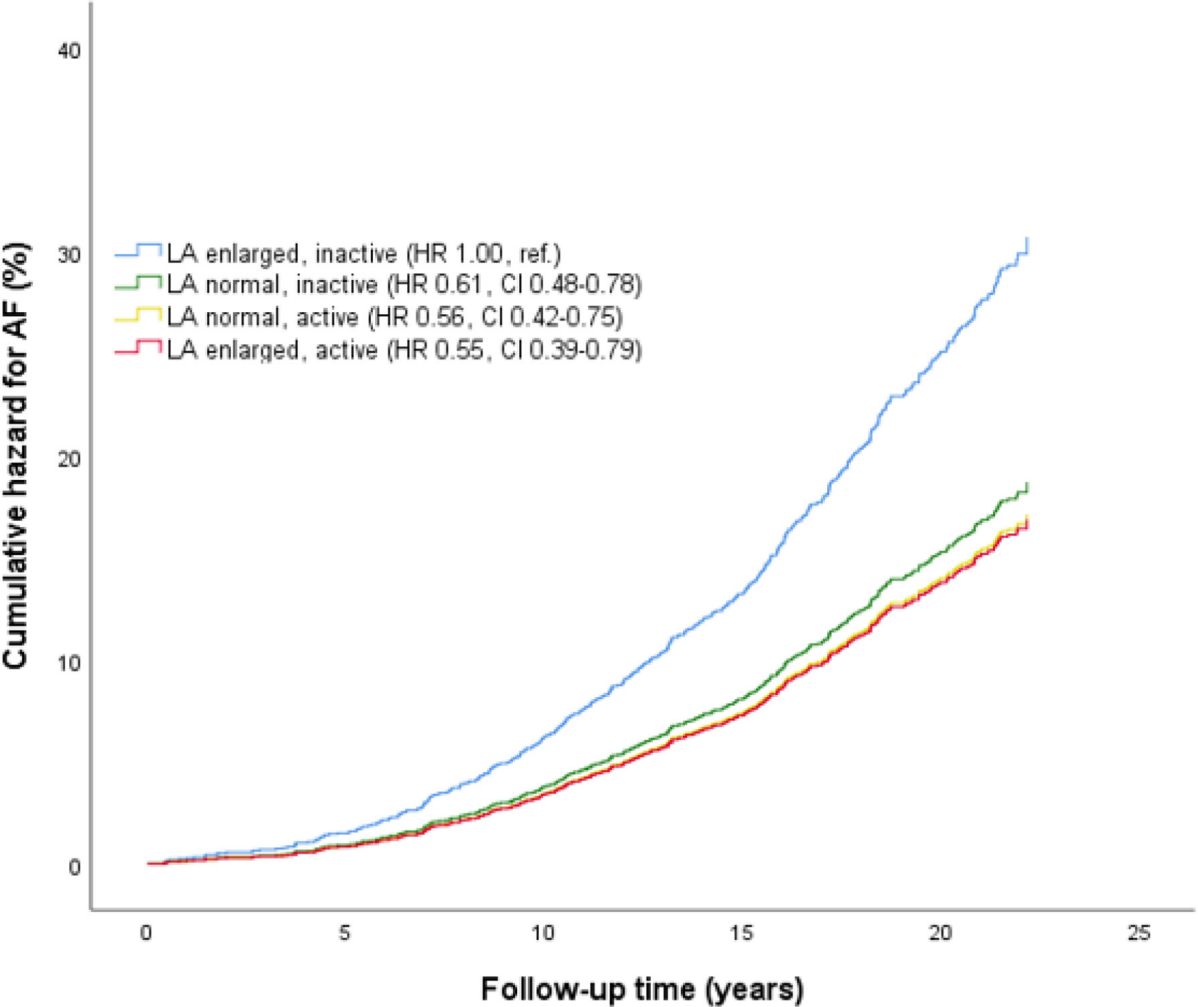
Adjusted cumulative hazard for AF by joint associations of PA and LA size with AF risk. Differences between groups are indicated with HR and 95% CI. The model is adjusted for age, sex, body mass index and systolic blood pressure. AF, atrial fibrillation, LA, left atrial/left atrium. The Tromsø Study 1994–1995.

In analyses stratified by LA size (table 3), we observed that PA was associated with lower risk of AF in participants with LA enlargement only, but significantly reduced only in the low PA group (HR_adjusted_ 0.41, 95% CI 0.22 to 0.77). Adjusted cumulative hazard for AF stratified by joint associations of PA and LA size is presented in figure 1. The increased AF risk with LA enlargement was attenuated by PA; compared with inactive participants with LA enlargement, the risk of AF was 45% lower among active with LA enlargement (HR_adjusted_ 0.55, 95% CI 0.39 to 0.79). Furthermore, the risk of AF in active participants with LA enlargement did not differ from participants with normal LA size (online supplemental table S1).

10.1136/openhrt-2021-001823.supp1 Supplementary data

**Table 3:** Associations between PA and AF stratified by LA size (HR ±95% CI): the Tromsø Study 1994–1995

	N (n=2479)	AF events, % (n)	Person-years (mean±SD)	Model 1, HR (95% CI)	Model 1, P-value	Model 2, HR (95% CI)	Model 2, P-value
**LA normal**	1714	13.6 (233)	17.0 (6.5)				
Inactive	1019	14.3 (146)	16.4 (6.7)	1.00 (ref.)		1.00 (ref.)	
Low	279	14.0 (39)	17.5 (6.6)	0.88 (0.62 to 1.26)	0.488	1.07 (0.75 to 1.54)	0.707
Moderate	286	10.1 (29)	18.2 (5.9)	0.61 (0.41 to 0.91)	0.015	0.73 (0.49 to 1.09)	0.125
Vigorous	130	14.6 (19)	17.9 (6.2)	0.91 (0.56 to 1.46)	0.681	1.05 (0.65 to 1.71)	0.837
**LA enlarged**	765	21.7 (166)	15.7 (6.6)				
Inactive	483	25.5 (123)	14.9 (6.7)	1.00 (ref.)		1.00 (ref.)	
Low	104	10.6 (11)	17.1 (6.2)	0.34 (0.18 to 0.63)	0.001	0.41 (0.22 to 0.77)	0.005
Moderate	105	18.1 (19)	16.8 (6.2)	0.60 (0.37 to 0.98)	0.041	0.62 (0.38 to 1.02)	0.061
Vigorous	73	17.8 (13)	17.5 (6.1)	0.56 (0.32 to 0.99)	0.047	0.62 (0.34 to 1.12)	0.113

Model 1 was unadjusted. Model 2 was adjusted for age, sex, body mass index and systolic blood pressure.

**Figure 2:**
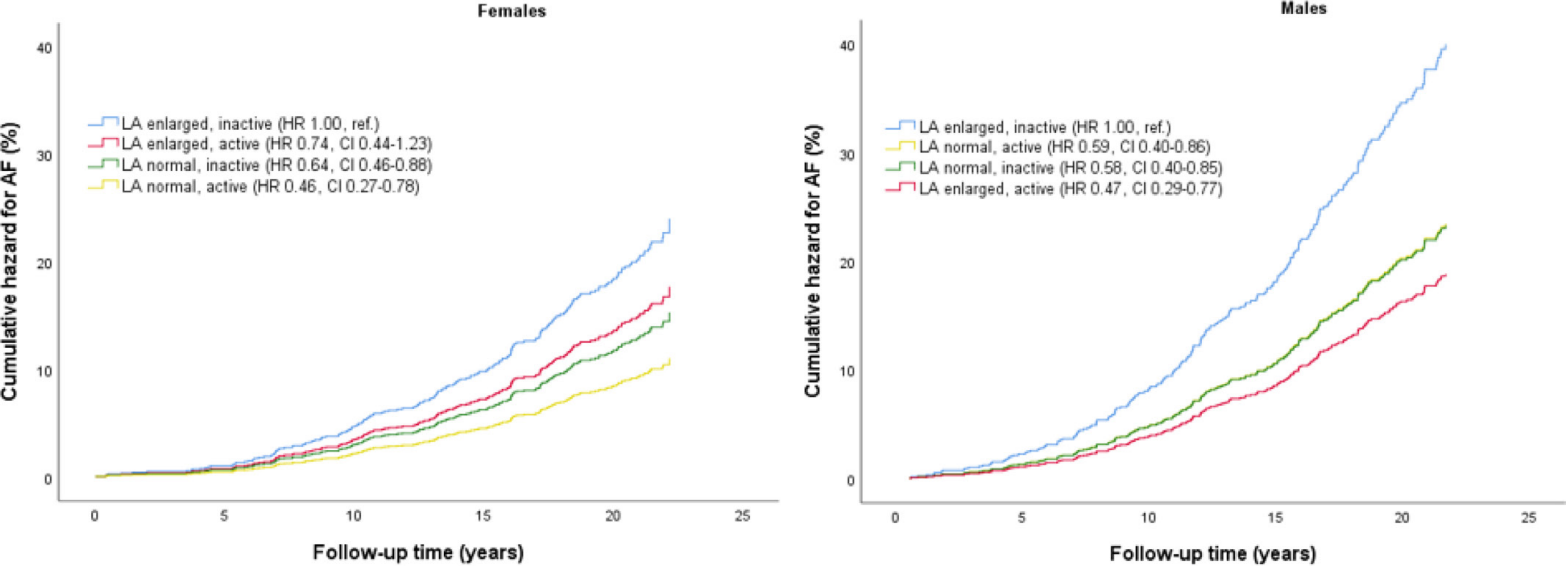
Adjusted cumulative hazard for AF by joint associations of PA and LA size with AF risk, stratified by sex. Differences between groups are indicated with HR and 95% CI. The model is adjusted for age, body mass index and systolic blood pressure. AF, atrial fibrillation, LA, left atrial/left atrium. The Tromsø Study 1994–1995.

PA, physical activity; LA, left atrial/left atrium; AF, atrial fibrillation; ref., reference.;

In sex stratified analyses, we observed 79% higher risk of AF in men than in women (HR_adjusted_ 1.79, 95% CI 1.47 to 2.19) (online supplemental table S2). Adjusted cumulative hazard for AF stratified by joint associations of PA and LA size, and sex, is presented in figure 2. In both women and men, active participants with LA enlargement had the same risk of AF as participants with normal LA size (online supplemental table S2).

**Figure 3:**
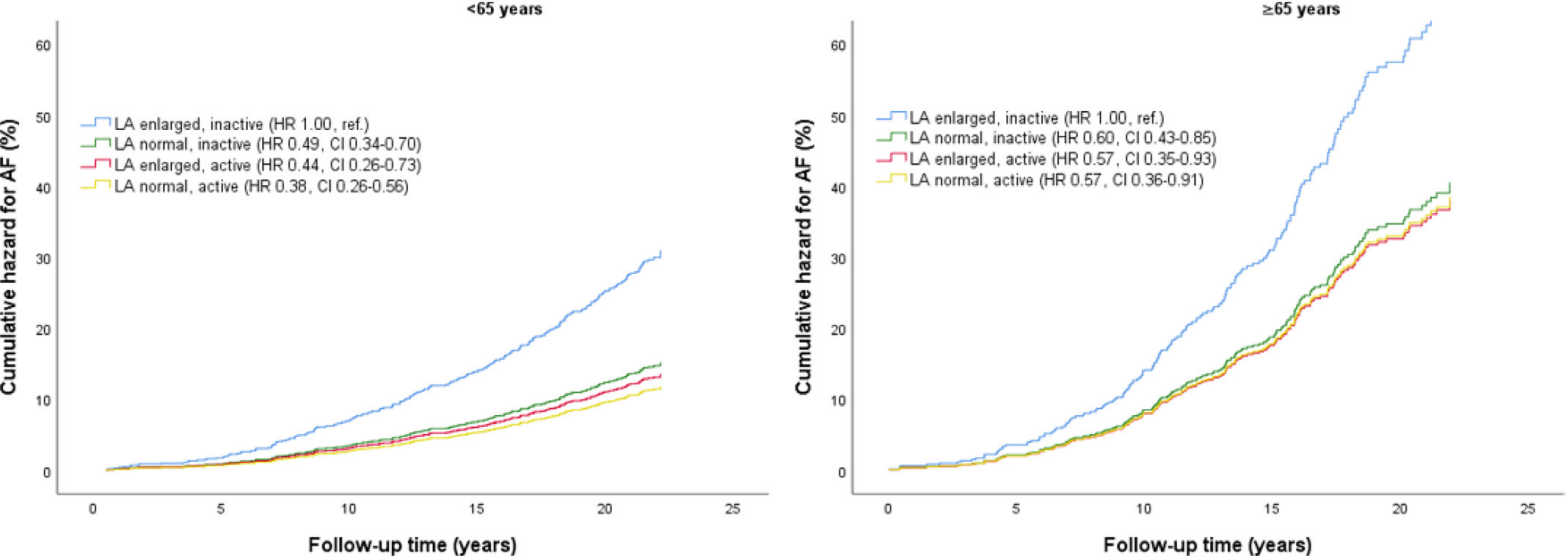
Adjusted cumulative hazard for AF by joint associations of PA and LA size with AF risk, stratified by age. Differences between groups are indicated with HR and 95% CI. The model is adjusted for sex, body mass index and systolic blood pressure. AF, atrial fibrillation, LA, left atrial/left atrium. The Tromsø Study 1994–1995.

When we stratified by age, participants ≥65 years had 2.6-fold higher risk of AF compared with participants <65 years (HR_adjusted_ 2.59, 95% CI 2.09 to 3.20) (online supplemental table S3). Adjusted cumulative hazard for AF stratified by joint associations of PA and LA size, and age, is presented in figure 3. The risk of AF in active with LA enlargement was not higher in any age groups compared with participants with normal LA size (online supplemental table S3).

Adjusted cumulative hazard for AF stratified by joint associations of PA, LA size and age, stratified by sex, is presented in online supplemental figure S1 and table S4). In general, men and women <65 years showed the lowest risk of AF compared with inactive ≥65 years with LA enlargement, which had the highest risk of AF. Moreover, in both men and women <65 years, the risk of AF was similar in active with LA enlargement as in those with normal LA size (online supplemental table S5).

### Sensitivity analysis

When we combined questions about light and hard PA into five categories (online supplemental table S6), the association between PA and AF was similar as in the main analyses with four levels of hard PA (table 2). Furthermore, the association between PA and AF did not change when we replaced systolic blood pressure (model 2) with hypertension groups (online supplemental table S7, model 3). Similarly, the association between PA and AF did not differ from model two when additional covariates (smoking, coffee consumption, diabetes, LDL cholesterol, palpitations, LV myocardial mass index, thyroid disease, C-reactive protein, alcohol consumption or resting heart rate) were added (online supplemental table S8, Model 3).

## Discussion

To the best of our knowledge, this is the first study that has analysed both PA and LA size jointly as risk factors for AF in a general population. The main findings from our study are that PA was associated with lower risk of AF in a U-shaped pattern, and the increased AF risk with LA enlargement was attenuated by PA. Furthermore, the risk of AF in active participants with LA enlargement did not differ from active with normal LA size. These patterns were observed in both men and women, and in participants over/under 65 years.

Therefore, in our study of participants free from known cardiac pathology, LA enlargement does not represent increased risk of AF in physically active individuals. Furthermore, inclusion of several risk factors for AF in the analysis did not influence the associations between PA, LA size and AF.

### Physical activity, LA size and AF

Overall, we observed 32% lower risk of AF in moderate active than in inactive participants, and a U-shaped association was seen between PA and risk of AF. The protective effect of PA on cardiovascular disease is well documented,^31^ and several studies support our observation that moderate doses of PA reduces the risk of AF.^3 5 32^ Also, our observation that the protective effect of PA on AF risk is offset by vigorous exercise, demonstrated by a U-shaped relationship, is consistent with previous studies.^5 6 33 34^ Furthermore, increased risk of AF is seen in endurance athletes,^7 9 33^ and with high levels of lifelong exercise.^8 34^ Our results showed that active participants had similar AF risk regardless of LA size, suggesting that LA enlargement in active individuals free from known cardiac pathology is not associated with increased risk of AF.

To our knowledge, no previous studies have examined how the relationship between LA size and AF varies with PA, and if PA attenuates the risk of AF seen with LA enlargement, in the general population. Our results, that LA enlargement increases the risk of AF, are consistent with previous research in both the general population^10 11^ and in healthy middle-aged individuals.^8^ Furthermore, we observed that moderate PA attenuated the risk of AF in participants with LA enlargement, whereas PA did not attenuate the risk of AF in participants with normal LA size.

The higher risk of AF observed in inactive with LA enlargement may be due to pathological conditions elevating chronic LA pressure and volume overload,^12^ for example, hypertension, LV diastolic dysfunction or heart failure, whereas the lower risk in active with LA enlargement may be due to the protective effect of moderate PA on modifiable cardiovascular risk factors.^35^ In our study, the participants were free from known cardiac pathology, which may explain why there was no beneficial association between PA and AF in participants with normal LA size.

Atrial stretch in response to LA volume and pressure overload is a key stimulus for LA enlargement and may potentially induce release of factors leading to atrial myocyte hypertrophy, oxidative stress and fibrosis, which increases the risk of AF.^12 35^ Although we excluded participants with known cardiac pathology at baseline, it is likely that the increased risk of AF in inactive with LA enlargement is explained by undetected pathological conditions we could not control for. Therefore, the reduced risk in active with LA enlargement may be the sum of the protective effect of moderate PA, outweighing the potentially increased risk of LA enlargement. However, the lower risk of AF in active with LA enlargement persisted even when we adjusted for multiple cardiovascular risk factors in our sensitivity analysis.

During vigorous exercise, atrial stretch may potentially release similar factors as in pathological conditions and thereby increase risk of AF,^12^ but an important difference between pathology and exercise is that the atrial pressure overload returns to normal when the exercise ceases.^12^ However, when repeated over time, and with insufficient recovery between exercise bouts, this may contribute to the increased risk of AF observed with high amounts of cumulative lifetime moderate-to-vigorous PA or exercise.^8 34 35^ Furthermore, it has been reported that male endurance athletes have a higher prevalence of masked hypertension (38%) than what is expected from the general population (8%–20%).^36^ Thus, masked hypertension might also explain the increased risk of AF associated with exercise.^37^ Other cardiac adaptations to vigorous exercise, associated with increased risk of AF, include increased vagal tone, resting bradycardia and electrical remodelling,^5 35^ which all may reduce atrial refractory period and facilitate re-entry.^12^

Cardiac chamber enlargement is assumed to be a physiological and reversible adaptation to exercise training.^35^ This adaptation is expressed as *the athlete’s heart*^35^ and is adapted to perfectly match the supply to the demands of exercise.^37^ However, it is suggested that LA enlargement itself may be a substrate for AF in athletes,^19 35^ and, therefore, that the athlete’s heart may potentially be proarrhythmic independent of other abnormalities.^37^

Interestingly, the pathophysiology seems to differ between ‘classical’ AF and exercise-related AF. AF is usually associated with high age, comorbidities and other risk factors.^1^ In contrast, people at risk of exercise-related AF are typically middle-aged men with athlete’s heart and high amounts of cumulative lifetime exercise.^1 19^ Moreover, this group typically has a lower prevalence of conventional risk factors for AF such as overweight and hypertension.^1^ However, little is known about the long-term risk associated with exercise-related AF,^19^ and longitudinal studies are needed to better understand the risk associated with exercise-related AF.

### Age and sex in the association between PA, LA size and AF

It is well documented that increasing age is a prominent risk factor for AF, and that men have higher risk of AF than women.^1^ In the Framingham Heart Study, the authors demonstrated a linear association between increasing age and risk of AF, and participants aged 60–69 years had fivefold risk of AF compared with participants aged 50–59.^38^ Moreover, men had twofold incidence of AF compared with women.^38^ Furthermore, the association between vigorous PA and AF differs between sexes, and higher risk of AF is seen in men, whereas women have lower risk of AF with high amounts of vigorous PA.^3 32 39^

In sex stratified analyses, we observed 79% higher risk of AF in men than in women, and when we stratified by age, participants ≥65 years had 2.6-fold higher risk of AF compared with participants <65 years. Furthermore, despite a higher overall risk of AF with LA enlargement, the risk of AF in active with LA enlargement was not higher in any sex, or age groups, compared with active participants with normal LA size.

### Strengths and limitations

Our study has several strengths. First, the prospective design with a long follow-up period in combination with echocardiography data. Second, hospital-diagnosed AF confirmed by electrocardiography and validated by an independent expert endpoint committee. Third, the large number of participants, the high attendance and the large age span strengthen our generalisability to other Caucasian populations. Fourth, as the University Hospital of North Norway is the only hospital within a large geographical region, most hospital confirmed AF cases in our study population are likely uncovered. Finally, the broad diversity of covariates allowed us to adjust for multiple potential confounders.

Our study has limitations that should be addressed. First, LA size was assessed by M-mode anteroposterior LA diameter, which is less accurate and have more geometrically assumptions than the recommended biplane calculated LA volume.^26^ However, a recent study demonstrated that anteroposterior LA diameter correlated well with LA volume (r=0.67), moreover, that there was high agreement (k=0.79) between LA volume and estimated LA volume by anteroposterior LA diameter in diagnosing LV diastolic dysfunction.^40^ Second, self-reported PA is prone to both recall and social desirability bias,^41^ although misclassification would probably have underestimated the true effects of PA. Third, as LA size was measured at baseline only, we cannot tell if the LA enlargement was due to exercise or pathology, or whether PA led to pathology and LA enlargement before baseline. However, the latter is less likely as we excluded all participants with present or previous known cardiac pathology, and we also adjusted for cardiovascular risk factors at baseline. Fourth, we cannot exclude residual confounding by measured or unmeasured variables (eg, masked hypertension, fibrosis or LA function). Finally, as AF was confirmed at the hospital, it is likely that some participants are misclassified as non-cases due to paroxysmal and/or silent AF that failed to be detected at examination. However, assuming that this misclassification is non-differential, the true AF prevalence may be higher than in our study, and the association between PA and AF is probably underestimated.

In conclusion, our prospective study of participants free from known cardiac pathology suggests a U-shaped relationship between PA and AF. Moderate PA was associated with reduced risk of AF, whereas vigorous PA attenuated the protective effect of moderate PA. Moreover, PA attenuated the increased AF risk with LA enlargement in both men and women, and in participants over/under 65 years. We suggest that the protective effect of moderate PA outweighs the potential risk of AF with LA enlargement. Further research is warranted to clarify the association between LA enlargement and AF in relation to PA, preferably assessed with biplane-derived LA volume and objective measures of PA.

## Data Availability

Data may be obtained from a third party and are not publicly available. The legal restriction on data availability is set by the Tromsø Study Data and Publication Committee in order to control for data sharing, including publication of datasets with the potential of reverse identification of deidentified sensitive participant information. The data can, however, be made available from the Tromsø Study upon application to the Tromsø Study Data and Publication Committee. Contact information: The Tromsø Study, Department of Community Medicine, Faculty of Health Sciences, UiT The Arctic University of Norway; e-mail: tromsous@uit.no.
