# Supplementary material for: Associations between physical activity, left atrial size and incident atrial fibrillation: the Tromsø Study 1994–2016
Source: Open Heart. 2022 Jan 24;9(1):e001823. doi: 10.1136/openhrt-2021-001823 (PMC8788327; doi:10.1136/openhrt-2021-001823)
Supplement: Supplementary data [file openhrt-2021-001823supp001.pdf]

SUPPLEMENTARY MATERIAL

Associations between physical activity, left atrial size, and incident atrial fibrillation: The Tromsø Study 1994-2016

Table S1. Joint associations of PA and LA size with AF (HR ±95% CI). The Tromsø Study 1994-95.

	N (n=2479)	AF events, % (n)	Person-years (mean ±SD)	Model 1, HR (95% CI)	Model 1, p-value	Model 2, HR (95% CI)	Model 2, p-value
LA enlarged, active (ref.)	282	15.2 (43)	17.1 (6.2)	1.00 (ref.)		1.00 (ref.)	
LA enlarged, inactive	483	25.5 (123)	14.9 (6.7)	2.03 (1.43-2.87)	<0.001	1.82 (1.28-2.59)	0.001
LA normal, inactive	1019	14.3 (146)	16.4 (6.7)	0.99 (0.70-1.39)	0.948	1.11 (0.79-1.56)	0.555
LA normal, active	695	12.5 (87)	17.9 (6.3)	0.76 (0.53-1.10)	0.148	1.02 (0.70-1.47)	0.938

PA: physical activity, LA: Left atrial, AF: atrial fibrillation, HR: Hazard ratio, CI: confidence interval, ref: reference, SD: standard deviation. Model 1 was unadjusted. Model 2 was adjusted for age, sex, body mass index, and systolic blood pressure.

Heitmann KA, *et al. Open Heart* 2022; 9:e001823. doi: 10.1136/openhrt-2021-001823

Table S2. Joint associations of PA and LA size with AF, stratified on sex (HR ±95% CI). The Tromsø Study 1994-95.

	N (n=2479)	AF events, % (n)	Person-years (mean ±SD)	Model 1, HR (95% CI)	Model 1, p-value	Model 2, HR (95% CI)	Model 2, p-value
**Female**	1298	14.3 (185)	17.2 (6.4)				
LA enlarged, active (ref.)	122	14.8 (18)	18.2 (5.6)	1.00 (ref.)		1.00 (ref.)	
LA enlarged, inactive	341	22.6 (77)	15.6 (6.7)	1.92 (1.15-3.20)	0.013	1.36 (0.81-2.27)	0.244
LA normal, inactive	584	12.3 (72)	17.1 (6.4)	0.91 (0.54-1.52)	0.714	0.86 (0.52-1.45)	0.581
LA normal, active	251	7.2 (18)	19.0 (5.7)	0.45 (0.24-0.87)	0.018	0.62 (0.32-1.21)	0.161
**Male**	1181	18.1(214)	15.9 (6.8)	1.41 (1.16-1.72) [a]	0.001 [a]	1.79 (1.47-2.19) [a]	<0.001 [a]
LA enlarged, active (ref.)	160	15.6 (25)	16.2 (6.4)	1.00 (ref.)		1.00 (ref.)	
LA enlarged, inactive	142	32.4 (46)	13.3 (6.5)	2.81 (1.72-4.57)	<0.001	2.12 (1.30-3.47)	0.003
LA normal, inactive	435	17.0 (74)	15.4 (6.9)	1.16 (0.74-1.83)	0.519	1.24 (0.78-1.95)	0.362
LA normal, active	444	15.5 (69)	17.2 (6.5)	0.91 (0.58-1.44)	0.682	1.24 (0.78-1.98)	0.354

PA: physical activity, LA: Left atrial, AF: atrial fibrillation, HR: Hazard ratio, CI: confidence interval, ref.: reference, SD: standard deviation. Model 1 was unadjusted. Model 2 was adjusted for age, body mass index, and systolic blood pressure. [a] = Overall difference from female group

Table S3. Joint associations of PA and LA size with AF, stratified on age (HR ±95% CI). The Tromsø Study 1994-95.

	N (n=2479)	AF events, % (n)	Person-years (mean ±SD)	Model 1, HR (95% CI)	Model 1, p-value	Model 2, HR (95% CI)	Model 2, p-value
**<65 years**	1697	12.7 (215)	17.9 (6.3)				
LA enlarged, active (ref.)	240	21.7 (52)	16.6 (6.6)	1.00 (ref.)		1.00 (ref.)	
LA enlarged, inactive	184	12.0 (22)	18.5 (5.8)	2.11 (1.28-3.48)	0.003	2.28 (1.37-3.80)	0.001
LA normal, inactive	690	11.7 (81)	17.6 (6.5)	1.04 (0.65-1.67)	0.858	1.12 (0.69-1.81)	0.642
LA normal, active	583	10.3 (60)	18.4 (6.1)	0.86 (0.53-1.40)	0.545	0.87 (0.53-1.42)	0.578
**≥65 years**	782	23.5 (184)	13.9 (6.4)	2.78 (2.28-3.40) [a]	<0.001 [a]	2.59 (2.09-3.20) [a]	<0.001 [a]
LA enlarged, active (ref.)	98	21.4 (21)	14.4 (6.0)	1.00 (ref.)		1.00 (ref.)	
LA enlarged, inactive	243	29.2 (71)	13.2 (6.4)	1.55 (0.95-2.51)	0.080	1.76 (1.07-2.90)	0.025
LA normal, inactive	329	19.8 (65)	13.8 (6.3)	0.98 (0.60-1.61)	0.946	1.07 (0.65-1.75)	0.802
LA normal, active	112	24.1 (27)	15.0 (6.5)	1.05 (0.59-1.86)	0.868	1.01 (0.57-1.79)	0.968

PA: physical activity, LA: Left atrial, AF: atrial fibrillation, HR: Hazard ratio, CI: confidence interval, SD: standard deviation, ref.: reference. Model 1 was unadjusted. Model 2 was adjusted for sex, body mass index, and systolic blood pressure. [a] = Overall difference from age group <65 years

Heitmann KA, *et al. Open Heart* 2022; 9:e001823. doi: 10.1136/openhrt-2021-001823

Table S4. Joint associations of PA, LA size and age with AF, stratified on sex. LA enlarged, inactive ≥65 years as reference. (HR ±95% CI). The Tromsø Study 1994-95

	N (n=2479)	AF events, % (n)	Person-years (mean ±SD)	Model 1, HR (95% CI)	Model 1, p-value	Model 2, HR (95% CI)	Model 2, p-value
**Female**	1298						
LA enlarged, inactive ≥65 years (ref.)	178	29.2 (52)	13.9 (6.6)	1.00 (ref.)		1.00 (ref.)	
LA enlarged, active ≥65 years	44	22.7 (10)	15.8 (6.0)	0.64 (0.33-1.27)	0.201	0.68 (0.34-1.33)	0.258
LA normal, active ≥65 years	40	17.5 (7)	17.2 (6.1)	0.42 (0.19-0.93)	0.033	0.53 (0.24-1.19)	0.123
LA normal, inactive ≥65 years	196	15.8 (31)	15.0 (6.1)	0.49 (0.31-0.76)	0.002	0.52 (0.33-0.80)	0.003
LA enlarged, inactive <65 years	163	15.3 (25)	17.5 (6.4)	0.36 (0.22-0.58)	<0.001	0.42 (0.26-0.67)	<0.001
LA normal, inactive <65 years	388	10.6 (41)	18.2 (6.3)	0.23 (0.15-0.35)	<0.001	0.31 (0.20-0.47)	<0.001
LA enlarged, active <65 years	78	10.3 (8)	19.6 (4.9)	0.20 (0.10-0.43)	<0.001	0.30 (0.14-0.63)	0.002
LA normal, active <65 years	211	5.2 (11)	19.3 (5.6)	0.11 (0.06-0.20)	<0.001	0.15 (0.08-0.29)	<0.001
**Male**	1181						
LA enlarged, inactive ≥65 years (ref.)	65	29.2 (19)	11.3 (5.7)	1.00 (ref.)		1.00 (ref.)	
LA normal, inactive ≥65 years	133	25.6 (34)	11.9 (6.2)	0.78 (0.45-1.37)	0.391	0.80 (0.46-1.40)	0.434
LA enlarged, inactive <65 years	77	35.1 (27)	14.9 (6.7)	0.75 (0.42-1.35)	0.335	0.73 (0.41-1.33)	0.306

LA normal, active ≥65 years	72	27.8 (20)	13.7 (6.4)	0.68 (0.36-1.28)	0.235	0.70 (0.37-1.31)	0.263
LA enlarged, active ≥65 years	54	20.4 (11)	13.3 (5.7)	0.54 (0.26-1.13)	0.103	0.57 (0.27-1.20)	0.140
LA normal, inactive <65 years	302	13.2 (40)	17.0 (6.6)	0.23 (0.13-0.40)	<0.001	0.23 (0.13-0.41)	<0.001
LA normal, active <65 years	372	13.2 (49)	17.9 (6.3)	0.21 (0.12-0.36)	<0.001	0.23 (0.13-0.40)	<0.001
LA enlarged, active <65 years	106	13.2 (14)	17.7 (6.3)	0.22 (0.11-0.43)	<0.001	0.22 (0.11-0.45)	<0.001

PA: physical activity, LA: Left atrial, AF: atrial fibrillation, HR: Hazard ratio, CI: confidence interval, SD: standard deviation, ref.: reference. Model 1 was unadjusted. Model 2 was adjusted for body mass index and systolic blood pressure

Heitmann KA, *et al. Open Heart* 2022; 9:e001823. doi: 10.1136/openhrt-2021-001823

Table S5. Joint associations of PA, LA size and age with AF, stratified on sex. LA enlarged, active <65 years as reference. (HR ±95% CI). The Tromsø Study 1994-95.

	N= (2479)	AF events, % (n)	Person-years (mean ±SD)	Model 2, HR (95% CI)	Model 2, p-value
**Female**	1298				
LA enlarged, active <65 years (ref.)	78	10.3 (8)	19.6 (4.9)	1.00 (ref.)	
LA enlarged, inactive ≥65 years (1)	178	29.2 (52)	13.9 (6.6)	3.39 (1.59-7.25)	0.002
LA enlarged, inactive <65 years (2)	163	15.3 (25)	17.5 (6.4)	1.41 (0.63-3.14)	0.402
LA enlarged, active ≥65 years (3)	44	22.7 (10)	15.8 (6.0)	2.29 (0.90-5.86)	0.083
LA normal, inactive ≥65 years (4)	196	15.8 (31)	15.0 (6.1)	1.74 (0.79-3.83)	0.166
LA normal, inactive <65 years (5)	388	10.6 (41)	18.2 (6.3)	1.04 (0.49-2.22)	0.919
LA normal, active ≥65 years (6)	40	17.5 (7)	17.2 (6.1)	1.80 (0.65-4.98)	0.255
LA normal, active <65 years (7)	211	5.2 (11)	19.3 (5.6)	0.50 (0.20-1.25)	0.137
**Male**	1181				
LA enlarged, active <65 years (ref.)	106	13.2 (14)	17.7 (6.3)	1.00 (ref.)	
LA enlarged, inactive ≥65 years (1)	65	29.2 (19)	11.3 (5.7)	4.46 (2.23-8.94)	<0.001
LA enlarged, inactive <65 years (2)	77	35.1 (27)	14.9 (6.7)	3.27 (1.71-6.26)	<0.001
LA enlarged, active ≥65 years (3)	54	20.4 (11)	13.3 (5.7)	2.55 (1.16-5.63)	0.021
LA normal, inactive ≥65 years (4)	133	25.6 (34)	11.9 (6.2)	3.56 (1.91-6.66)	<0.001
LA normal, inactive <65 years (5)	302	13.2 (40)	17.0 (6.6)	1.04 (0.56-1.93)	0.900
LA normal, active ≥65 years (6)	72	27.8 (20)	13.7 (6.4)	3.11 (1.57-6.18)	0.001
LA normal, active <65 years (7)	372	13.2 (49)	17.9 (6.3)	1.02 (0.56-1.86)	0.943

PA: physical activity, LA: Left atrial, AF: atrial fibrillation, HR: Hazard ratio, CI: confidence interval, SD: standard deviation, ref.: reference. Model 1 was unadjusted. Model 2 was adjusted for body mass index and systolic blood pressure

Table S6. Association between PA with five categories and AF (HR ±95% CI). The Tromsø Study 1994-95.

	N (n=2472)	AF events, % (n)	Person-years (mean ±SD)	Model 1, HR (95% CI)	Model 1, p-value	Model 2, HR (95% CI)	Model 2, p-value
Sedentary [a]	265	17.7 (47)	15.2 (6.8)	1.00 (ref.)		1.00 (ref.)	
Inactive [b]	1236	18.0 (222)	16.1 (6.7)	0.94 (0.68-1.28)	0.682	1.00 (0.73-1.38)	0.987
Low [c]	381	13.1 (50)	17.4 (6.5)	0.61 (0.41-0.91)	0.014	0.80 (0.54-1.21)	0.290
Moderate [d]	390	12.3 (48)	17.8 (6.0)	0.56 (0.37-0.83)	0.004	0.68 (0.45-1.03)	0.068
Vigorous [e]	200	15.5 (31)	17.7 (6.1)	0.71 (0.45-1.11)	0.131	0.85 (0.54-1.36)	0.500

PA: physical activity, AF: atrial fibrillation, HR: Hazard ratio, CI: confidence interval, SD: standard deviation, ref.: reference. Model 1 was unadjusted. Model 2 was adjusted for sex, body mass index, and systolic blood pressure.

[a] = light and hard PA 0 hours/week

[b] = light PA >0 hours/week and hard PA 0 hours/week

[c] = hard PA 0-1 hour/week

[d] = hard PA 1-2 hours/week

[e] = hard PA ≥3 hours/week

Heitmann KA, *et al. Open Heart* 2022; 9:e001823. doi: 10.1136/openhrt-2021-001823

Table S7. Sensitivity analysis: Association between PA and AF adjusted for hypertension groups (HR ±95% CI). The Tromsø Study 1994-95.

	N (n=2468)	AF events, % (n)	Model 1, HR (95% CI)	Model 2, HR (95% CI)	Model 3, HR (95% CI)
Inactive	1497	18.0 (269)	1.00 (ref.)	1.00 (ref.)	1.00 (ref.)
Low	382	13.1 (50)	0.64 (0.47-0.87)	0.80 (0.59-1.09)	0.79 (0.58-1.08)
Moderate	388	12.1 (47)	0.58 (0.42-0.79)	0.67 (0.49-0.92)	0.68 (0.50-0.93)
Vigorous	201	15.9 (32)	0.76 (0.53-1.10)	0.89 (0.61-1.29)	0.88 (0.61-1.28)

PA: physical activity, AF: atrial fibrillation, HR: Hazard ratio, CI: confidence interval, ref.: reference.

Model 1 was unadjusted. Model 2 was adjusted for age, sex, body mass index, and systolic blood pressure.
Model 3 was adjusted for age, sex, body mass index, and hypertension groups.

Heitmann KA, *et al. Open Heart* 2022; 9:e001823. doi: 10.1136/openhrt-2021-001823

Table S8. Sensitivity analysis: Association between PA and AF with covariates in Model 3 (HR ±95% CI). The Tromsø Study 1994-95.

	N (n=1614)	AF events, % (n)	Model 1, HR (95% CI)	Model 2, HR (95% CI)	Model 3, HR (95% CI)
Inactive	872	16.1 (140)	1.00 (ref.)	1.00 (ref.)	1.00 (ref.)
Low	293	14.0 (41)	0.80 (0.56-1.13)	0.95 (0.66-1.35)	0.95 (0.66-1.37)
Moderate	298	10.7 (32)	0.60 (0.41-0.88)	0.65 (0.44-0.97)	0.66 (0.45-0.98)
Vigorous	151	16.6 (25)	0.95 (0.62-1.45)	1.01 (0.65-1.56)	0.95 (0.61-1.47)

PA: physical activity, AF: atrial fibrillation, HR: Hazard ratio, CI: confidence interval, ref.: reference, LDL: low-density lipoprotein, LV: left ventricular.

Model 1 was unadjusted. Model 2 was adjusted for age, sex, body mass index, systolic blood pressure. Model 3 was adjusted for Model 2 in addition to smoke, coffee, diabetes, LDL cholesterol, palpitations, LV myocardial mass index, thyroid disease, C-reactive protein, alcohol, resting heart rate.

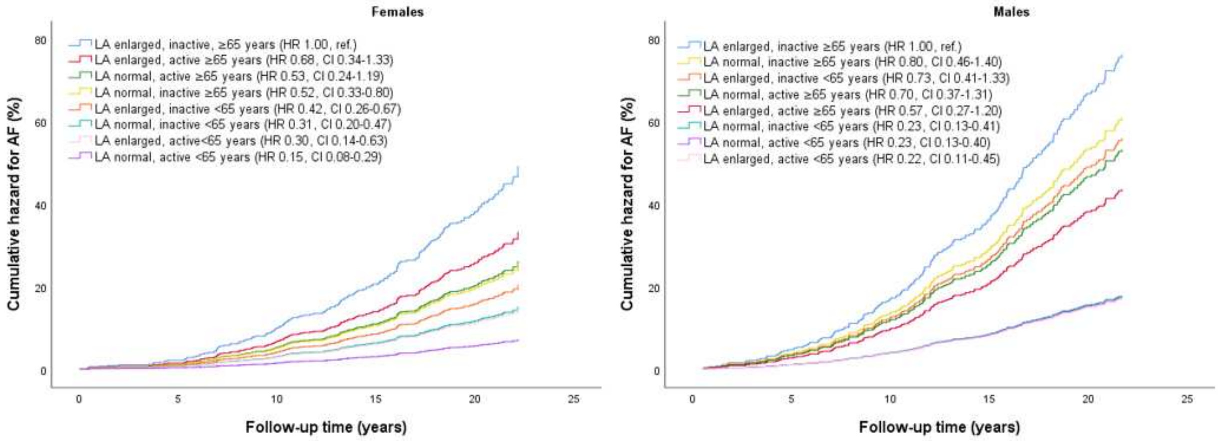

Figure S1. Adjusted cumulative hazard for AF by joint associations of PA, LA size, and age with AF risk, stratified by sex. Differences between groups are indicated with HR and 95% CI. The model is adjusted for body mass index and systolic blood pressure. AF: atrial fibrillation, LA: left atrial/left atrium, HR: hazard ratio, CI: confidence interval. The Tromsø Study 1994-95.

Heitmann KA, *et al. Open Heart* 2022; 9:e001823. doi: 10.1136/openhrt-2021-001823
